# Case reports: three novel variants in PCCA and PCCB genes in Chinese patients with propionic acidemia

**DOI:** 10.1186/s12881-020-01008-y

**Published:** 2020-04-06

**Authors:** Qi Yang, Hong Xu, Jingsi Luo, Mengting Li, Sheng Yi, Qinle Zhang, Guoxing Geng, Shihan Feng, Xin Fan

**Affiliations:** 1 Department of Genetic and Metabolic Central Laboratory, Guangxi Maternal and Child Health Hospital, No.59, Xiangzhu Road, Nanning, China; 2 NanNing Region Center for Disease Prevention and Control, No.55, Xiangzhu Road, Nanning, China

**Keywords:** Propionic acidemia (PA), Chromatography-mass spectrometry(GC-MS), Tandem mass spectrometry (MS/MS), PCCA and PCCB genes, Mutation

## Abstract

**Background:**

Propionic acidemia (PA) is an autosomal recessive metabolic disorder caused by the deficiency of the mitochondrial protein propionyl-CoA carboxylase (PCC) and is associated with pathogenic variants in either of the two genes *PCCA* or *PCCB*. The present study aimed to identify the genetic cause of three Chinese patients with PA.

**Case presentation:**

Three Chinese PA patients were diagnosed by using gas chromatography-mass spectrometry(GC-MS), tandem mass spectrometry (MS/MS) and molecular diagnostic methods. All patients had onset in the neonatal period. One patient died of infection and metabolic decompensation, and the other two had mild to moderate developmental delay/mental retardation. Mutation analysis of the *PCCA* gene identified that patient 1 carried the compound heterozygous c.1288C > T(p.R430X) and c.2002G > A(p.G668R), and patient 2 was homozygous for the c.1426C > T(p.R476X) mutation. Mutation analysis of the *PCCB* gene identified that patient 3 harbored the compound heterozygous mutations c.359_360del AT(p.Y120Cfs*40) and c.1398 + 1G > A. Among these mutations, three (c.1288C > T, c.359_360del AT and c.1398 + 1G > A) are novel.

**Conclusions:**

We reported three Chinese PA patients who had *PCCA* or *PCCB* mutants. Among them, in the *PCCA* gene, c.1288C > T(p.R430X) was a nonsense mutation, resulting in a truncated protein. c.359_360del AT was a frameshift mutation, leading to a p.Y120Cfs*40 change in the amino acid sequence in the PCCB protein. c.1398 + 1G > A was a splicing mutation, causing skipping of the exons 13–14. In conclusion, the novel mutations uncovered in this study will expands the mutation spectrum of PA.

## Background

Propionic academia (PA) is one of most common organic acidemias. It is inherited in an autosomal recessive fashion, and is caused by the deficiency of the mitochondrial protein propionyl-CoA carboxylase ((PCC; EC 6.4.1.3), which catalyzes the carboxylation of propionyl CoA to yield D-methylmalonyl-CoA [1]. Patients with PA often present with acute deterioration, cardiac arrhythmias, metabolic acidosis, dyspnea and hyperammonemia. Without prompt diagnosis and management, patients can deteriorate quickly and lapse into lethargy, seizures, or sudden death [2].

PCC, a mitochondrial biotin-dependent enzyme in the catabolic pathway of amino acids [3], is composed of an α6β6 structure [4]. The α and β subunits are encoded by *PCCA* (MIM 232000) and *PCCB* (MIM 232050) genes localized on chromosomes 13q32 and 3q21-q22, respectively [5, 6]. To date, more than 200 mutations in both the *PCCA* and *PCCB* genes have been reported (www.hgmd.cf.ac.uk/ac/gene.). There is a large degree of genetic heterogeneity in the *PCCA* gene, and no prevalent mutations have been observed in any population studied. On the contrary, in the *PCCB* gene, there was a limited number of mutations accounting for most of the patients in different ethnic groups. Among the Japanese, c.923-924insT, c.1644-6C > G and R399Q are the most common mutations [7]. There are two main types of *PCCB* gene mutations in patients with PA in Latin America, c.1218-1231dell4insl2 and E168K, accounting for more than 60% of cases [8]. Here we reported three patients with PA who were diagnosed using gas chromatography-mass spectrometry (GC-MS) and tandem mass spectrometry (MS/MS) [9]. We then identified three novel mutations in the *PCCA* and *PCCB* genes by Sanger sequencing.

## Case presentation

### Affected individuals

Three unrelated patients are from Guangxi China, and were clinically diagnosed with PA using via urine organic acid analysis by gas chromatography-mass spectrometry (GC-MS) and carnitine analysis by tandem mass spectrometry (MS/MS) (Table 1).

**Table 1:** The results of MS/MS and GC/MS in neonatal period

DATE	Time	MS/MS				GC/MS
		C0 (10–50 μmol/L)	C3 (0.38–3.6 μmol/L)	C3/C2 (0.04–0.2)	C3/C0 (0.02–0.17)	3-Hydroxypropionate (0–1.1)
Patient 1	At the time of diagnosis	4.62↓	8.99↑	1.60↑	1.94↑	63.52↑
	–	–	–	–	–	–
	–	–	–	–	–	–
Patient 2	At the time of diagnosis	1.55↓	7.19↑	5.48↑	4.65↑	280.39↑
	After 1 month of treatment	3.22↓	5.54↑	3.21↑	1.72↑	70.55↑
	Latest	4.23↓	6.13↑	1.82↑	1.45↑	30.21↑
Patient 3	At the time of diagnosis	13.1	9.46↑	0.77↑	0.72↑	26.41↑
	After 3 month of treatment	14.5	7.34↑	0.65↑	0.51↑	24.63↑
	Latest	13.59	7.26↑	0.81↑	0.63↑	25.12↑

### Clinical phenotype

All patients were born full term with normalbirth weight. Patient 1 was a boy and Patient 3 was a girl both of non-consanguineous parents. Patient 2 was a boy of first-degree-cousin parents. All patients were screened in the Genetic and Metabolic Central Laboratory affiliated to Guangxi Maternal and Child Health Hospital. The results are shown in Table 1. Propionyl glycine, 3-hydroxypropionate and methyl citrate were increased in urine excretion. MS/MS showed increased of acylcarnitine profile revealed and increased C3-acylcarnitin, C3/C2 and C3/C0 acylcarnitine ratio.

Patient 1 was 3 weeks old when he presented with tachypnea and malignant hyperthermia, and was diagnosed with presumed sepsis. He was admitted to the hospital three times due to recurrent andrespiratory tract infections. At 6 months of age, he developed generalized tonic clonic seizures, metabolic acidosis (bicarbonate, 15.4 mmol/L) and hyperammonemia (ammonia, 516 μmol/L). Only then did he undergo the screening by MS/MS and GC-MS, and diagnosed with PA. Unfortunately, he died at six and a half months of age from sudden cardiac arrest.

Patient 2 had suffered from recurrent vomiting, lethargy and hyperventilation at the age of 1 month. Fortunately, the patient was diagnosed with PA and treated with low-isoleucine, −methionine, −threonine, and-valine diet, L-carnitine, and biotin at the first time. Thereafter, he experienced a few metabolic crises; and the result of MS/MS and GC/MS detection showed an improvement in metabolic level (Table 1). He underwent fundoplication and gastrostomy tube placement at age of 1.5 years for caloric intake. He is now 3 years old and has shown mild mental retardation, and his weight has increased from the 5th percentile to the 20th percentile.

Patient 3 was screened on the third day after birth. She initially received an intravenous infusion, low isoleucine, methionine, threonine and proline special formulations, as well as oral carnitine and biotin treatment. Thereafter, she had several episodes of infections, diarrhea, metabolic acidosi; and the result of MS/MS and GC/MS detection are shown in Table 1. She is now 5 years old with moderate mental retardation. In the past few years, she has been admitted to the hospital several times due to episodes of infections,diarrhea, metabolic acidosis and generalized tonic-clonic seizures. She also had poor weight gain (6th percentile). Her recent heart assessment is normal.

### Mutation analysis

Peripheral blood was obtained from the patients and their parents. DNA was isolated from peripheral blood using the Lab-Aid DNA kit (Zeesan Biotech Co., Ltd., Xiamen, China) according to the manufacturer’s protocol. NanoDrop ND-2000 spectrophotometer and software (NanoDrop 2000; NanoDrop Technologies; Thermo Fisher Scientific, Inc., Waltham, MA, USA) were used for DNA quality detection. Primer version 3 (frodo.wi.mit.edu) was used to design PCR primers for PCR amplification of all exons and and flanking introns of the PCCA (NM_000282.3) and PCCB genes (NM_000532.4) (Tables 2 and 3). Each 50 μl primary PCR mixture contained 200 ng genomic DNA, 2.5 μl 10X buffer, 8 μl of a dNTP mixture (2.5 mmol/L), 2 U Taq DNA polymerase (Takara Biotechnology Co., Ltd., Dalian, China), 1.5 μl (10 μmol/L) of each prime. PCR amplification including the following steps: (1) denaturing initial: 95 °C for 5 min. (2) 35 cycles of 95 °C for 30 s, 56~60 °C for 30 s, 72 °C for 60 s, (3) final extension: 72 °C for 10 min. The PCR products were sequenced directly in an ABI 3500 genetic analyzer. (Thermo Fisher Scientific, Inc.).

**Fig. 1:**
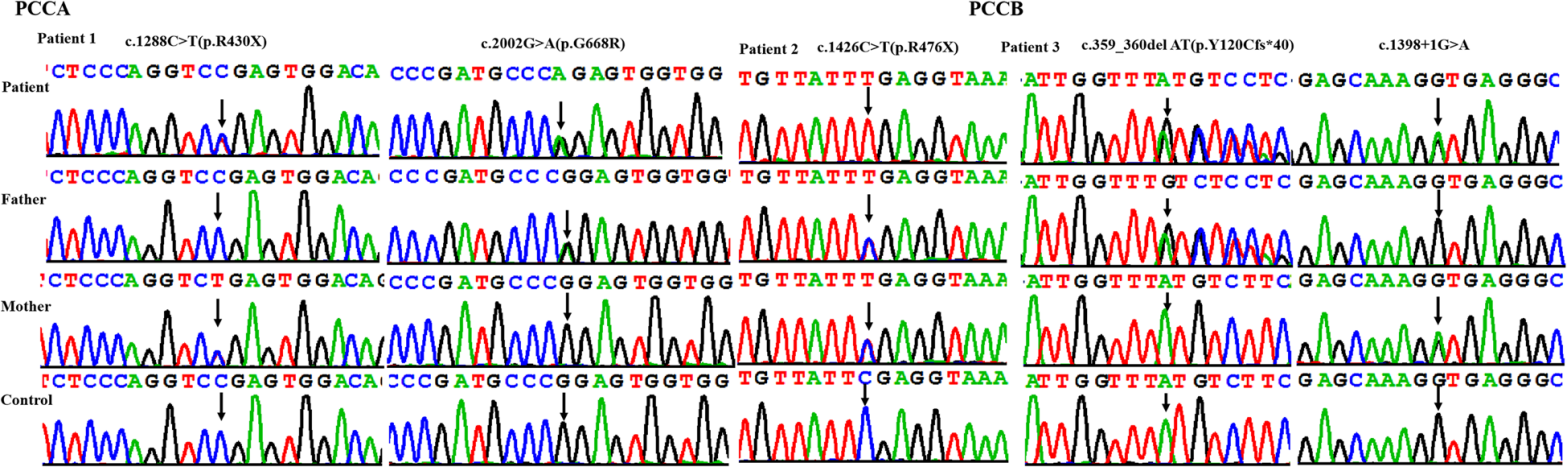
DNA sequencing result from PCCA (Patient1–2) and PCCB (Patient3) gene, changes also seen in the father or/ and mother, but not seen in a representative normal subject. Three novel mutations: c.1288C > T(p.R430X) (PCCA, patientv1); c.359_360delAT(p.Y120Cfs*40) (PCCB, patient 3), c.1398 + 1G > A (PCCB, patient 3)

To evaluate whether novel variants were disease-causing mutations or polymorphisms, PolyPhen 2.0 and Mutation Taster tools were performed to analyze the functional effects of novel variants. Variants were further evaluated according to the ACMG and AMP standards and guidelines [10]. Three *PCCA* mutations and two *PCCB* mutations were identified. Patient 1 was compound heterozygous for c.1288C > T and c.2002G > A changes in the *PCCA* gene. Heterozygous c.1288C > T and c.2002G > A mutations were identified in the father and mother, respectively. Patient 2 was homozygous for c.1426C > T(p.R476X) in the *PCCA* gene, and his parents were heterozygous. Patient 3 was compound heterozygous for c.359_360del AT and c.1398 + 1G > A changes in the *PCCB* gene. The heterozygous c.359_360del AT and c.1398 + 1G > A mutations were identified in the father and mother, respectively. The novel mutations of the *PCCA* gene(c.1288C > T) and *PCCB* gene(c.359_360del AT and c.1398 + 1G > A) were not detected in any of the 200 normal controls (600 alleles) enrolled in this study. According to the ACMG standards and guidelines for the interpretation of sequence variants, these novel mutations are pathogenic. Clinical findings and genotypes are summarized in Table 4 and Fig. 1.

**Table 2:** PCR primers and conditions used for mutation analysis of the *PCCA* gene

Exon	Sequence(5′-3′)			
	**Forword**	**Reverse**	**Product size(bp)**	**Annealing temperature(°C)**
1	ACTAGCCCTCCAGGTCCTAG	GGAAAGCAAGCGGTGTAGC	612	62
2	TATTGCCTAGAACTACATTTATTGA	ACAGTGTGGAGACGAGAAAGT	276	58
3–4	AACTTGGTGTTTTTTGGTCTTAAA	GTATGTCTCACTTTTTCTGCTTG	362	58
5	TACGACTCTATAAATGATAGGCA	CCTTTGATTTCCAGTAGCGAAT	395	58
6	AAGTGTACTTATTCAAGGGCTC	AATTAAAATTCTATAATCCATCACTA	273	56
7	GTGTTGGCTCAAAAACTGTTGT	TGTGTGTGTGTGTGTATATCCC	380	60
8	ATGAATCGGAGGAGACAGTAG	CTTCCAGAGCAACAAGTAATCA	271	58
9	ATTATTGTTTTCTGCGTTATTGAAC	CATTATGCTTTTGGTATCTGTTTAC	266	58
10	GTCTGACTCTTCTTCTCCTTCTTC	TCTAAAGGCACAACTCACAATCA	293	60
11	AAATAATGTTTTGAGAGGTATGTATA	CAACAGAGTGAGACCCTGTCT	347	60
12	AACTTTAAGAAAATGTTTATGTAATG	ATCTTTATTTAACACTTTATGGAGT	349	60
13	TGTGATTTTTCTTGTTTGTTTCTAT	TACCTCATTGTTTGGCTATACC	295	60
14	TTGATTTTTAAGTACATTCTAAGTTG	TCACTTGTCCTTCAATTTACACC	365	60
15	TTTTCCTATTTTCCAGAAGTTGAA	ACTACAAACTAACATAACGCTGAA	393	60
16	ATGTATTGAAAACTGAACTATCATAA	AATGTGCTGTGCCATATTCTCA	315	60
17	TTTATTGATACCACAAGTTCAGATA	TCAAAATAGAGTGAAATTATGTATTAC	362	60
18	AATAGATGCCCTATAAAATACTTG	ACTTCTCCAAAGACCCATAAGAT	338	60
19	GATGATTCTTAGAGTAGGTGTTTA	GATGAAAAATACATAGAGGTACTAC	367	60
20	AAAATGGCTGCTGCTTTGTATG	CATTAAATGCTCAGGTTAAAACTTA	371	60
21	GTTTTTTGGCTATCGTGAACATTA	CCTAGAATATCATTTGTAAAGGCA	272	60
22	TGATTTAGAATGAATGCTACTTTTGA	CTGTTTAGGGGGCGTCGGT	345	60
23	CACATATTTTGGGGCATTTGACA	GTAGAAGCGAGGGGGAGAGG	304	60

**Table 3:** PCR primers and conditions used for mutation analysis of the *PCCB* gene

Exon	Sequence(5′-3′)			
	**Forword**	**Reverse**	**Product size(bp)**	**Annealing temperature(°C)**
1	TGGTCAGAGAAGAGCAAGGAC	GGTTATACCCGCCTCCACG	603	60
2	GCCCTTGCTTTGCTTACTAAAT	AGTGTACGGTCGCTCACGC	449	60
3	GCCAAACTCATTAGAAGAAGTATT	TTTTCCCCAAACTACAAGCAAGT	240	56
4	CTGTCTCCAGGGCTCAAGCAA	CCGCAAAGATACTCAATAAGCAC	447	60
5	CTATTAAATATCTGGTCCTTTGTC	ACAATGCGGCAGAGAACAATG	410	58
6	TGTTATCTTATTGTTGTCTTTATCT	ACACCTTATCATCACTATGCTG	332	58
7	GCTGAATCAACTCTAAGGCTGT	CAGTCTTCCCAAATAAGGTCTG	301	60
8	AAGGTATGTATTATGTGGCATTAC	AAGGTATGTATTATGTGGCATTAC	350	60
9	CGTGTCACCCCATTTCCTTTC	CGTGTCACCCCATTTCCTTTC	249	58
10	AGTGTCATTACATCTTATACTTGTC	ATTTAGATTTTTTCCTCTGGTCAT	314	56
11–12	GGATGGCTGCTGAGGACAAAT	CTGCGGGGCTGGGAACAAC	691	60
13	TAGGGCTATTCTTGTTCTTTGTC	GGAGACTTACCAAGGTCTAGGT	358	60
14	CCCACACACAGTGATAATGAGTT	CAAAGGCAAAAAGGCAGTTATGT	276	60
15	CTGAGCAGAAGGTTGAGGGGT	ATTAGGAGATAGATAGGGCATATT	639	60

**Table 4:** Summary of the clinical features and genotypes of the patients with propionic acidemi

Patient NO	Gender	Mutations		Onset Diagnosis	Current age	Outcome
		Paternal	Maternal			
PCCA						
1	M	c.2002G > A(p.G668R)	c.1288C > T(p.R430X)	21d	Died at 6.5 m	Epilepsy, possible cardiomyopathy
2	F	c.1426C > T(p.R476X)	c.1426C > T(p.R476X)	1 mon	3 y	Mild mental retardation, normal growth
PCCB						
3	M	c.359_360delAT (p.Y120Cfs*40)	c.1398 + 1G > A	3d	5y	Moderate mental retardation, epilepsy, growth failure

## Discussion and conclusions

In China, increasing numbers of patients with genetic metabolic deficiencies are being diagnosed and treated in the neonatal period [11]. However, in terms of managing PA patients, it is different from those countries where newborn screening for these disorders is freely available. Some cases will only seek medical attention if intoxication has appeared. In this study, although all PA cases presented in an acute neonatal form, patient 1 and 2 were diagnosed with PA, after presenting clinical symptoms, and not through neonatal screening. Patient 1 initially presented with tachypnea and malignant hyperthermia, and was diagnosed with presumed sepsis. At that time, the patient’s relevant metabolic levels (such as blood ammonia level) were not measured. This resulted in a delay in reaching until an accurate diagnosis, during which the patient developed generalized tonic clonic seizures, metabolic acidosis and hyperammonemia. The patient died at six and a half months of age from sudden cardiac arrest. Patient 2 was diagnosed by metabolite screening at the first time after symptoms appeared. After treatment, the prognosis was good. Newborn screening is a very important means of reducing the burden and the mokrtality rate of the disease [12].

In this study, we identified three different mutations in the *PCCA* gene and two different mutations in the *PCCB* gene. Patient 1 was compound heterozygous for c.1288C > T and c.2002G > A changes in the *PCCA* gene. The c.2002G > A(p.G668R) mutation has been previously reported [13], that is maps to the biotinylation domain, and it is defective in biotin binding [14]. The other mutation c.1288C > T has been reported in the Genome Aggregation Database as a SNP (rs776821944); however, the frequency of the variant only in genome aggregation was 0.000008/1, and was reported to be of no clinical significance. The mutation of c.1288C > T (p.R430X) is located in biotin carboxylation domain of the PCC enzyme and causes a premature termination codon, resulting in a truncated protein that undergoes nonsense-mediated decay (NMD) [15, 16]. According to the ACMG standards and guidelines for the interpretation of sequence variants, the mutation of c.1288C > T(p.R430X) is pathogenic. The homozygous mutation c.1426C > T(p.R476X) in the *PCCA* gene identified in Patient 2 was firstly reported in Indian patients [2]. Deepti Gupta et al. demonstrated that the nonsense mutation c.1426C > T(p.R476X) could form truncated proteins that undergo NMD [17]. The mutations of c.2002G > A(p.G668R), c.1288C > T (p.R430X) and c.1426C > T(p.R476X) impact the structure of the α subunit and reduced the activity of the PCC enzyme to varying degrees, leading to a variable phenotypes in patients. Patient 3 was compound heterozygous for c.359_360delAT and c.1398 + 1G > A mutations in the *PCCB* gene. To the best of our knowledge, c.359_360delAT(p.Y120Cfs*40) and c.1398 + 1G > A in the *PCCB* gene are novel mutations. These mutants were not present in the HGMD (http://www.ncbi.nlm.nih.gov), ClinVar(http://www.hgmd.cf.ac.uk/ac/), dbSNP (http://www.ncbi. nlm.nih.gov/SNP) or the 1000 Genomes databases (http://browser.1000genomes.org/index.html). The variant c.359_360delAT(p.Y120Cfs*40) caused a frameshift alteration after codon 120 leading to a premature termination codon (PTC) which is located at codon 160, resulting in truncation of the PCCB protein, thus, leading to a loss of function. A similar splicing mutation (c.1398 + 1G > T) has been reported to be associated with PA. The splicing mutation c.1398 + 1G > T in the *PCCB* gene results in exons 13–14 being skipped, leading to a novel aberrant aberrant transcripts [17]. We propose that our novel variant (c.1398 + 1G > A) acts in the same manner. The splice site mutation (c.1398 + 1G > A) is expected to eliminate splicing following exon 13, and causes exons 13–14 skipping, leading to aberrant splicing of the transcript. The CoA carboxyl transferase N-terminal and C-terminal form the active site of the PCCB subunit where c.359_360delAT(p.Y120Cfs*40) and c.1398 + 1G > T are part of the active site and thus predicted to affect the catalysis or substrate binding. Therefore, according to the ACMG standards and guidelines for the interpretation of sequence variants, the mutations of c.359_360delAT(p.Y120Cfs*40) and c.1398 + 1G > A are pathogenic. To date, of all mutations in PCCB described in patients, phenotypic severity has been observed to differ in patients with biallelic nonsense, deletion, or null mutations, which may be related to differences between individuals in NMD activity and NMD efficiency. Splice site variants are also seen, and, in general, result in milder disease [18]. Therefore, our patients exhibit a varying degrees of phenotype, which may be due to differences in protein activity, expression, and timely treatment.

In summary, we report 3 PA patients and the molecular basis of their disease were identified by PCR-sequencing of all coding exons of the PCCA and PCCB genes. Three novel mutations, c.1288C > T (p.R430X**)** in the PCCA gene, c.359_360delAT(p.Y120Cfs*40) and c.1398 + 1G > A in the PCCB gene, were identified. The present study will expand the mutation spectrum of PA.

## Data Availability

The datasets generated and analysed during the current study are available in the Mendeley repository, https://data.mendeley.com/datasets/dk3pfbb8f5/3 (DOI: 10.17632/dk3pfbb8f5.3).

## Abbreviations

PA: Propionic acidemia
GC-MS: Gas chromatography-mass spectrometry
MS/MS: Tandem mass spectrometry
PCC: Propionyl-CoA carboxylase
